# Increased memory B cell potency and breadth after a SARS-CoV-2 mRNA boost

**DOI:** 10.1038/s41586-022-04778-y

**Published:** 2022-04-21

**Authors:** Frauke Muecksch, Zijun Wang, Alice Cho, Christian Gaebler, Tarek Ben Tanfous, Justin DaSilva, Eva Bednarski, Victor Ramos, Shuai Zong, Brianna Johnson, Raphael Raspe, Dennis Schaefer-Babajew, Irina Shimeliovich, Mridushi Daga, Kai-Hui Yao, Fabian Schmidt, Katrina G. Millard, Martina Turroja, Mila Jankovic, Thiago Y. Oliveira, Anna Gazumyan, Marina Caskey, Theodora Hatziioannou, Paul D. Bieniasz, Michel C. Nussenzweig

**Affiliations:** 1 Laboratory of Retrovirology, The Rockefeller University, New York, NY USA; 2 Laboratory of Molecular Immunology, The Rockefeller University, New York, NY USA; 3 Howard Hughes Medical Institute, The Rockefeller University, New York, NY USA

**Keywords:** Antibodies, RNA vaccines, Immunological memory, SARS-CoV-2

## Abstract

The Omicron variant of SARS-CoV-2 infected many vaccinated and convalescent individuals^1–3^. Despite the reduced protection from infection, individuals who received three doses of an mRNA vaccine were highly protected from more serious consequences of infection^4^. Here we examine the memory B cell repertoire in a longitudinal cohort of individuals receiving three mRNA vaccine doses^5,6^. We find that the third dose is accompanied by an increase in, and evolution of, receptor-binding domain (RBD)-specific memory B cells. The increase is due to expansion of memory B cell clones that were present after the second dose as well as the emergence of new clones. The antibodies encoded by these cells showed significantly increased potency and breadth when compared with antibodies obtained after the second dose. Notably, the increase in potency was especially evident among newly developing clones of memory cells, which differed from persisting clones in targeting more conserved regions of the RBD. Overall, more than 50% of the analysed neutralizing antibodies in the memory compartment after the third mRNA vaccine dose neutralized the Omicron variant. Thus, individuals receiving three doses of an mRNA vaccine have a diverse memory B cell repertoire that can respond rapidly and produce antibodies capable of clearing even diversified variants such as Omicron. These data help to explain why a third dose of a vaccine that was not specifically designed to protect against variants is effective against variant-induced serious disease.

## Abstract (web summary)

A third dose of an mRNA vaccine against SARS-CoV-2 results in an expanded B cell repertoire that produces antibodies with increased potency and breadth.

## Main

We studied the immune responses to severe acute respiratory syndrome coronavirus 2 (SARS-CoV-2) mRNA vaccination in a longitudinal cohort of 42 volunteers with no prior history of SARS-CoV-2 infection^5,6^ who were recruited between 21 January 2021 and 14 December 2021 for sequential blood donation. The volunteers received either the Moderna (mRNA-1273; *n* = 8) or Pfizer-BioNTech (BNT162b2; *n* = 34) mRNA vaccine. Volunteers ranged in age from 23 to 78 years old; 48% were male and 52% were female (Methods and Supplementary Table 1). Samples were obtained at the following time points: 2.5 weeks after the prime, 1.3 and 5 months after the second vaccine dose and 1 month after the third dose.

## Plasma binding and neutralization

**Fig. 1:**
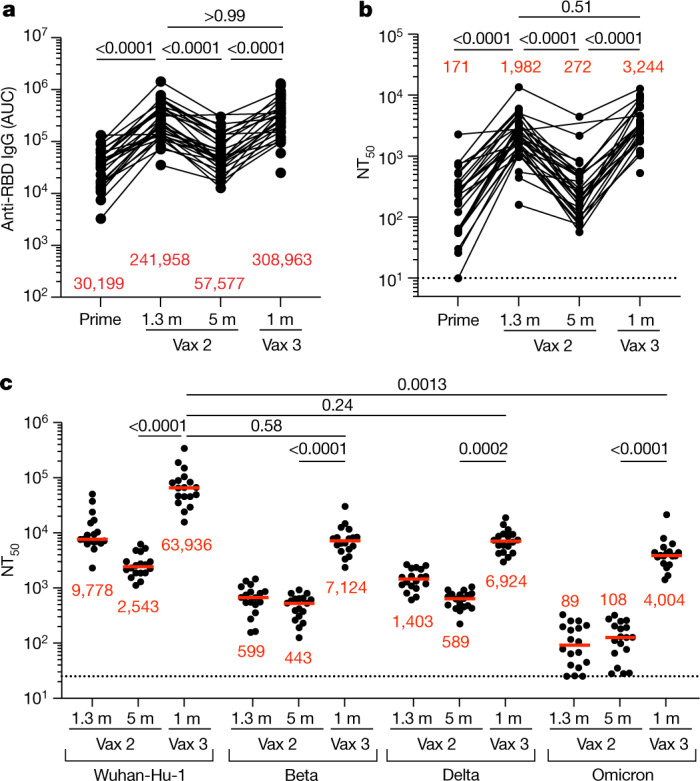
Plasma ELISAs and neutralizing activity. **a**, Graph showing area under the curve (AUC) for plasma IgG antibody binding to SARS-CoV-2 Wuhan-Hu-1 RBD after prime^6^, 1.3 months and 5 months after the second vaccination (Vax 2)^5,6^ and 1 month after the third vaccination booster (Vax 3) for *n* = 42 samples. Lines connect longitudinal samples. m, month. **b**, Graph showing anti-SARS-CoV-2 NT_50_ values of plasma measured by a SARS-CoV-2 pseudotype virus neutralization assay in 293T_Ace2_ cells using wild-type (WT; Wuhan-Hu-1)^32^ SARS-CoV-2 pseudovirus^16,33^ in the plasma samples shown in **a**. **c**, Plasma neutralizing activity against the indicated SARS-CoV-2 variants of interest or concern for *n* = 15 randomly selected samples assayed in HT1080Ace2cl.14 cells. Wuhan-Hu-1 and Omicron BA.1 NT_50_ values were derived from ref. ^7^. Pseudoviruses in **c** were based on a spike protein that also included the R683G substitution, which disrupts the furin cleavage site and increases particle infectivity. All experiments were performed at least in duplicate. Red bars and values in **a**–**c** represent geometric mean values. Statistical significance in **a** and **b** was determined by two-tailed Kruskal–Wallis test with subsequent Dunn’s multiple-comparisons test. Statistical significance in **c** was determined by two-tailed Friedman test with subsequent Dunn’s multiple-comparisons test. *P* values are as indicated.

**Extended Data Fig. 1:**
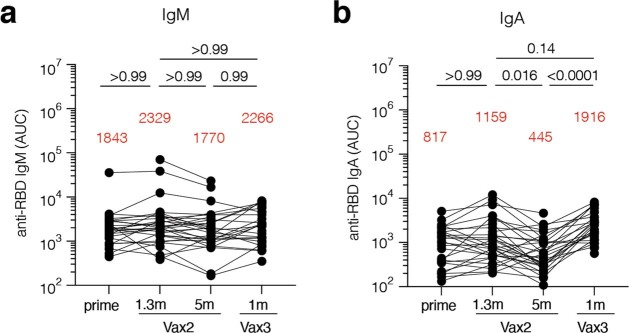
Plasma ELISA. Graph shows area under the curve (AUC) for plasma **a**, IgM and **b**, IgA antibody binding to Wuhan-Hu-1 SARS-CoV-2 RBD after prime^6^, 1.3 months (m) and 5 months (m) after the 2^nd^ vaccine dose (Vax2)^5,6^, and 1 month after the 3^rd^ (Vax3) for n = 42 samples. Lines connect longitudinal samples. Red bars and value represent geometric mean values. Statistical significance was determined by two-tailed Kruskal-Wallis test with subsequent Dunn’s multiple comparisons. P-values as indicated.

Plasma IgM, IgG and IgA responses to SARS-CoV-2 Wuhan-Hu-1 receptor-binding domain (RBD) were measured by enzyme-linked immunosorbent assay (ELISA)^5,6^. While a significant decrease was observed in antibody reactivity at 5 months after the second vaccine dose, anti-RBD IgG titres were significantly increased after a third dose of an mRNA vaccine (*P* < 0.0001) (Fig. 1a and Supplementary Table 1). The resulting titres were similar to those observed 1.3 months after the second dose (*P* > 0.99) (Fig. 1a). IgM and IgA titres were lower than IgG titres, and, although IgM titres were unchanged during the observation period, IgA titres were also significantly increased after the third vaccine dose (Extended Data Fig. 1 and Supplementary Table 1).

The plasma neutralizing activity in 42 participants was measured using HIV-1 pseudotyped with Wuhan-Hu-1 SARS-CoV-2 spike protein^5,6^ (Fig. 1b and Supplementary Table 1). While a 7.3-fold decrease in neutralizing titres occurred between 1.3 and 5 months after the second vaccine dose, administration of a third vaccine dose boosted neutralizing titres 11.9-fold, resulting in a geometric mean half-maximal neutralizing titre (NT_50_) of 3,244 against Wuhan-Hu-1 (Fig. 1b). Plasma neutralizing antibodies elicited by mRNA vaccination are more potent against Wuhan-Hu-1 than variants^5,6^. Consistent with prior reports^3,7–10^, the third vaccine dose significantly boosted geometric mean NT_50_ values by 16-fold, 12-fold and 37-fold for the Beta, Delta and Omicron BA.1 variants, respectively. The level of activity against the Beta and Delta variants was not significantly different from that against Wuhan-Hu-1, whereas the activity against Omicron BA.1 was 16-fold lower than that against Wuhan-Hu-1 (*P* = 0.58, *P* = 0.24 and *P* = 0.0013, respectively) (Fig. 1c). The correlates of protective neutralizing titres against Omicron BA.1 are not defined. Nevertheless, given the correlation between neutralizing antibody levels and protection from Wuhan-Hu-1 infection^11–13^, the reduced activity against Omicron BA.1 in recipients of a third dose of vaccine probably explains why vaccinated individuals remained particularly susceptible to infection by this variant.

## Memory B cells

**Fig. 2:**
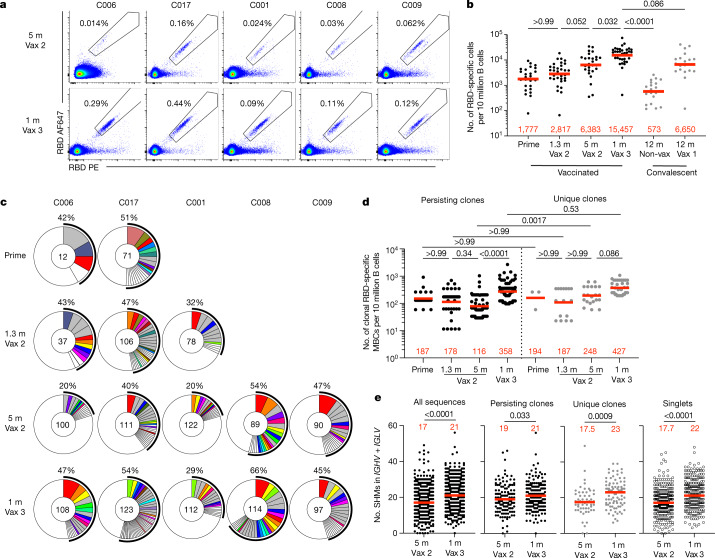
Anti-SARS-CoV-2 RBD memory B cells after a third vaccination. **a**, Representative flow cytometry plots showing dual binding of AF647- and PE-labelled RBD from Wuhan-Hu-1 by single sorted B cells from five individuals 5 months after the second vaccine dose^6^ and 1 month after the third vaccine dose (additional gating strategy given in Extended Data Fig. 2). The percentage of Wuhan-Hu-1 RBD-specific B cells is indicated. **b**, Graph summarizing the number of Wuhan-Hu-1 RBD-specific memory B cells per 10 million B cells after prime^5,6^, 1.3 and 5 months after the second vaccine dose^5,6^ and 1 month after the third vaccine dose (*n* = 42) compared with convalescent infected individuals 12 months after infection with or without later vaccination^15^ (grey dots). **c**, Pie charts showing the distribution of IgG antibody sequences obtained for memory B cells from five individuals after prime^6^, 1.3 and 5 months after the second vaccine dose^5,6^ and 1 month after the third vaccine dose. Time points are indicated to the left of the charts. The number inside the circle indicates the number of sequences analysed for the individual denoted above the circle. The pie slice size is proportional to the number of clonally related sequences. The black outline and associated numbers indicate the percentage of clonal sequences detected at each time point. Coloured slices indicate expanded persisting clones found at multiple time points within the same individual, grey slices indicate expanded clones unique to the time point and white slices indicate sequences isolated only once per time point. **d**, Graph showing the number of clonal Wuhan-Hu-1 RBD-specific memory B cells per 10 million B cells isolated from five participants. Each dot represents one clone illustrated in **c** (*n* = 331). The left panel (black dots) represents persisting clones, and the right panel (grey dots) represents time point-unique clones. **e**, Number of nucleotide somatic hypermutations (SHM) in *IGHV* and *IGLV* in all sequences detected 5 months after the second vaccine dose^6^ (*n* = 512) or 1 month after the third vaccine dose (*n* = 554) compared with the somatic hypermutations in *IGHV* and *IGLV* for sequences from persisting clones, unique clones and singlets. Red bars and numbers represent geometric mean values (**b**, **d**) or median values (**e**). Statistical difference was determined by two-tailed Kruskal–Wallis test with subsequent Dunn’s multiple-comparisons test (**b**, **d**) or by two-tailed Mann–Whitney test (**e**). *P* values are as indicated.

**Extended Data Fig. 2:**
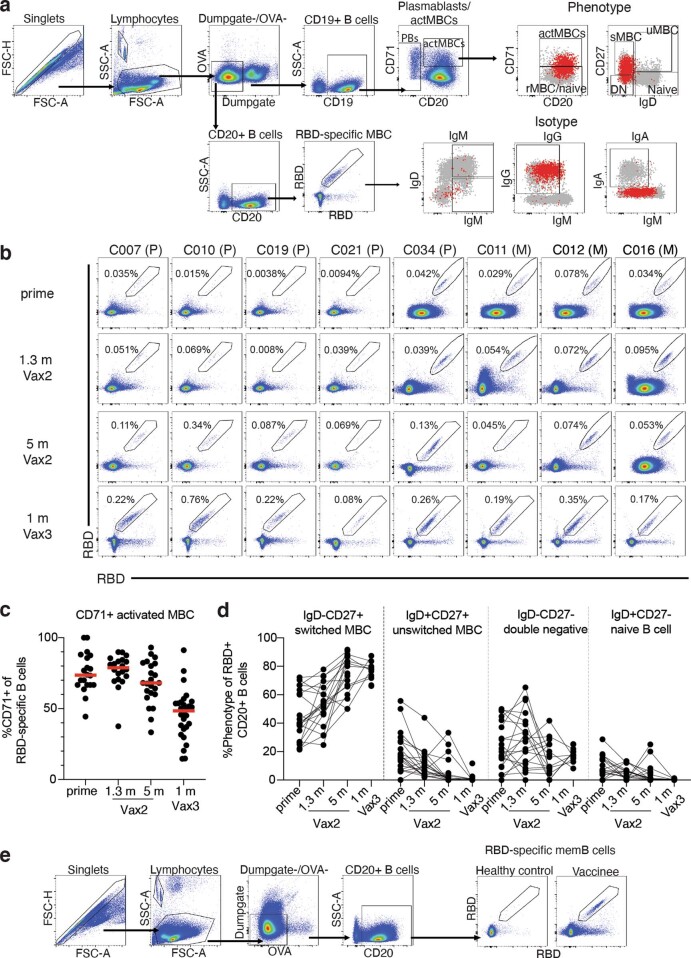
Flow Cytometry. **a**, Gating strategy for phenotyping. Gating was on lymphocytes singlets that were CD19^+^ or CD20^+^ and CD3-CD8-CD16-Ova-. Anti-IgG, IgM, IgA, IgD, CD71 and CD27 antibodies were used for B cell phenotype analysis. Antigen-specific cells were detected based on binding to Wuhan-Hu-1 RBD-PE^+^ and RBD-AF647^+^. Counting beads were added to each sample and gated based on forward scatter (FSC) and side scatter (SSC) as per manufacturer instructions. **b**, Representative flow cytometry plots of Wuhan-Hu-1-RBD-specific memory B cells in 8 individuals after prime^6^, 1.3- and 5-months post-Vax2^5,6^, and 1 month after Vax3. Timepoint of sample collection indicated to the left. Pfizer vaccinees indicated by (P) and Moderna by (M) across the top. **c**, Graph showing frequency of Wuhan-Hu-1-RBD-specific MBCs expressing activation marker CD71 over time after vaccination for n = 36 samples. Red bar indicated median value. **d**, Graph showing the phenotype of RBD-specific B cells over time, determined to be either switched MBCs (IgD-CD27+), unswitched MBCs (IgD+CD27+), double negative MBCs (IgD-CD27-) or naïve B cells (IgD+CD27-), for n = 18 samples. Lines connect longitudinal samples. **f**, Gating strategy for single-cell sorting for CD20+ memory B cells for Wuhan-Hu-1 RBD-PE and Wuhan-Hu-1 RBD-AF647.

Under physiological conditions, memory B cells produce little if any secreted antibody. However, when challenged with antigen as in a breakthrough infection, these cells undergo clonal expansion and produce antibody-secreting plasma cells and memory and germinal centre B cells^14^. To examine the effects of the third vaccine dose on the memory compartment in our longitudinal cohort, we performed flow cytometry experiments using phycoerythrin (PE)- and Alexa-Fluor-647 (AF647)-labelled Wuhan-Hu-1 RBDs (Fig. 2a and Extended Data Fig. 2). Individuals who received a third vaccine dose developed significantly increased numbers of Wuhan-Hu-1 RBD-binding memory cells compared with those who received only two doses or who were naturally infected^5,6,15^ (Fig. 2a, b). The number of memory cells produced after the third dose was not significantly higher than for vaccinated convalescent individuals (*P* = 0.08) (Fig. 2b). An increased proportion of memory B cells circulating after the third dose expressed IgG and lower levels of CD71 (Extended Data Fig. 2c).

**Extended Data Fig. 3:**
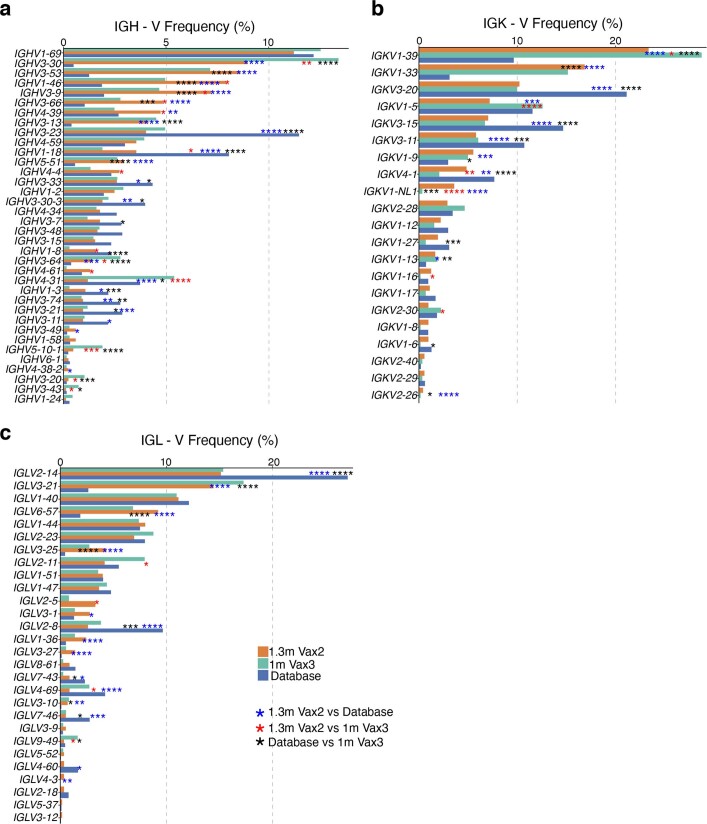
Frequency distribution of human V genes. **a-c** Comparison of the frequency distribution of human V genes for heavy chain and light chains of anti-RBD Wuhan-Hu-1 antibodies from this study and from a database of shared clonotypes of human B cell receptor generated by Cinque Soto et al.^40^. Graph shows relative abundance of human *IGHV* (**a**), *IGKV* (**b**) and *IGLV* (**c**) genes Sequence Read Archive accession SRP010970 (blue), 1.3m-Vax 2 antibodies (orange), and 1m-Vax3 antibodies (green).

We obtained 1,370 paired antibody sequences from five individuals who were sampled 5 months after the second vaccine dose and 1 month after the third vaccine dose. Two and three of these participants were additionally sampled 2.5 weeks after the first dose and 1.3 months after the second dose, respectively^5,6^ (Fig. 2c and Supplementary Table 2). After the third vaccine dose, all individuals examined showed expanded clones of memory B cells that were either persisting clones of memory B cells of the same clonal family detected at two or more time points after the initial vaccination or unique clones of memory B cells detected at only a single time point (Fig. 2c). Clones observed uniquely after the third vaccine dose could represent entirely new lineages elicited by the boost or memory cells that were present below the limit of detection at earlier time points. As at earlier time points, the *IGHV3*-*30*, *IGHV3*-*53* and *IGHV4*-*31* genes were over-represented 1 month after the third dose^5,6^ (Extended Data Fig. 3). Thus, there is a persistent bias in *IGHV* gene representation in memory that is common to most individuals.

**Extended Data Fig. 4:**
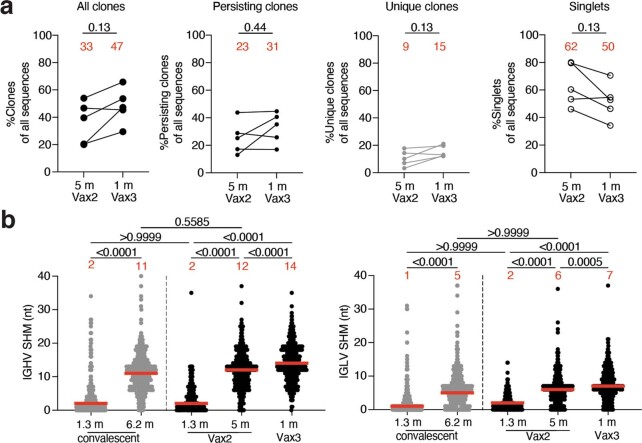
Clonality and somatic hypermutation of anti-SARS-CoV-2 Wuhan-Hu-1 RBD antibody clones after third vaccination booster. **a**, Graphs show relative fraction of all clones (persisting and unique), persisting clones only (sequences detected at multiple time points), unique clones only (clonally expanded but detected at a single time point), and singlets (detected only once at a single time point) among all antibody sequences in individuals (n = 5) 5m after the 2^nd^ and 1 month after the 3^rd^ dose. **b**, Number of nucleotide somatic mutations (SHM) in the *IGHV* (left panel) and *IGLV* (right panel) in the antibodies illustrated in Fig. 2c for vaccinees after 1.3- and 5- months post-Vax2^5,6^ and 1 month after Vax3, compared to the number of mutations obtained after 1.3^16^ or 6.2^17^ months after infection (grey). Statistical significance was determined by Wilcoxon matched-pairs signed rank test (**a**) or by two-tailed Kruskal Wallis test with subsequent Dunn’s multiple comparisons (**b)**. P-values as indicated.

Expanded clones of memory cells accounted for 33% and 47% of the repertoire 5 months after the second dose and 1 month after the third dose, respectively (Fig. 2c and Extended Data Fig. 4a). The relative increase in clonality was due in part to an average 3.1-fold expansion of persisting Wuhan-Hu-1 RBD-specific memory B cells (*P* < 0.0001) (Fig. 2d). Consistent with the relatively modest number of additional cell divisions by persisting clones, these clones accumulated on average only two additional somatic mutations, suggesting a germinal centre-independent process^14^ (Fig. 2e and Extended Data Fig. 4b).

In comparison with the increase observed in the number of newly emerging unique clones of memory cells at 5 months after the second dose, the increase in these clones after the third dose was more modest (1.7-fold) and did not reach statistical significance (*P* = 0.086) (Fig. 2d). Antibodies from these cells were more mutated than those from the unique clones present 5 months after the second vaccine dose, as were antibodies that were represented only once (singlets). In both cases, the numbers of somatic mutations were significantly greater than those observed 5 months after the second dose, indicating persisting evolution and cell division (*P* = 0.0009 and *P* < 0.0001, respectively) (Fig. 2e and Extended Data Fig. 4). Therefore, a third mRNA vaccine dose is associated with expansion and further evolution of the memory B cell compartment.

## Monoclonal antibodies

**Extended Data Fig. 5:**
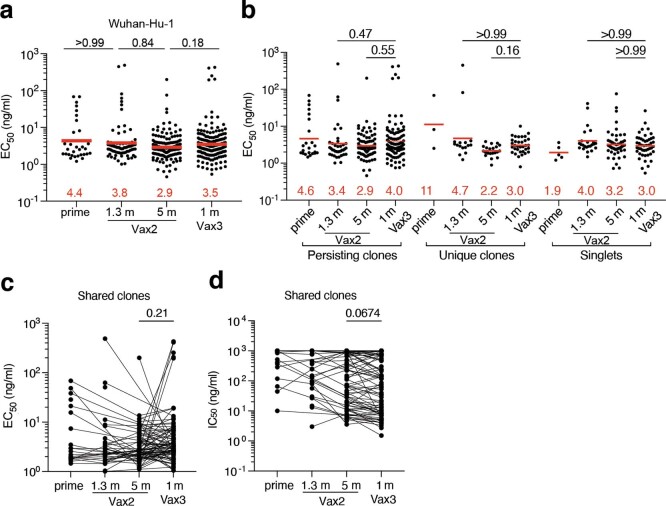
Anti-SARS-CoV-2 RBD monoclonal antibodies. **a**, Graphs show half-maximal concentration (EC_50_) of n = 459 monoclonal antibodies measured by ELISA against Wuhan-Hu-1 RBD after prime^6^, 1.3- and 5-months post-Vax2^5,6^, and 1 month after Vax3. **b**, Graph showing EC50 of n = 459 monoclonal antibodies as categorized as either persisting clones detected at multiple time points, unique clones where sequences were clonally expanded but detected at a single time point, or singlets where mAbs were derived from sequences detected once at a single time point. **c-d**, EC_50_ (**c**) or IC_50_ neutralizing activity (**d**) of n = 246 monoclonal antibodies derived from shared clones only. Lines connect the related clones at the indicated time point. Red bars and numbers in **a**, and **b**, indicate geometric mean values. Statistical significance was determined by two-tailed Kruskal Wallis test with subsequent Dunn’s multiple comparisons. All experiments were performed at least twice.

We cloned the sequences for and expressed 472 monoclonal antibodies, including 1 representative monoclonal antibody from each clonally expanded family and at least 9 randomly selected monoclonal antibodies from individual memory B cells detected only once in each participant (Supplementary Table 3). When tested by ELISA, 459 of these antibodies bound to Wuhan-Hu-1 RBD, indicating the high efficiency of the RBD-specific memory B cell isolation method used here (Extended Data Fig. 5 and Supplementary Table 3). Moreover, 191 antibodies obtained after the third vaccine dose were compared with 34 antibodies isolated after the prime as well as with 79 and 168 antibodies isolated 1.3 and 5 months after the second vaccine dose, respectively. Overall, there was no significant change in binding over time or with the number of vaccine doses (Extended Data Fig. 5a and Supplementary Table 3). This was true for all antibodies combined as well as for persisting clones detected at multiple time points, unique clones detected at only a single time point and single antibodies (Extended Data Fig. 5a–c).

**Fig. 3:**
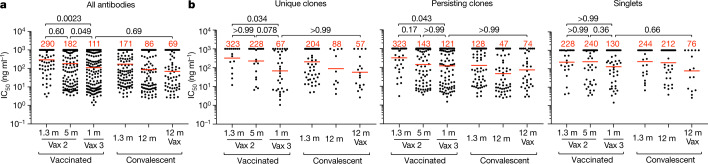
Anti-SARS-CoV-2 RBD monoclonal antibodies. **a**, **b**, Graphs showing the anti-SARS-CoV-2 neutralizing activity of monoclonal antibodies measured by a SARS-CoV-2 pseudotype virus neutralization assay using WT (Wuhan-Hu-1)^32^ SARS-CoV-2 pseudovirus^16,33^. IC_50_ values are shown for all antibodies (**a**) and for monoclonal antibodies categorized as unique clones (sequences clonally expanded but detected at a single time point), persisting clones (sequences detected at multiple time points) or singlets (monoclonal antibodies derived from sequences detected once at a single time point) (**b**). Antibodies were from vaccinated individuals 1.3 and 5 months after the second vaccine dose^5,6^ and 1 month after the third vaccination, convalescent individuals 1.3 months^16^ or 12 months^15^ after infection or vaccinated convalescent individuals 12 months after infection. Each dot represents 1 antibody; 459 total antibodies were tested, including the 325 reported herein (Supplementary Table 4) and 134 previously reported^6^. Red bars and numbers indicate geometric mean values. Statistical significance was determined by two-tailed Kruskal–Wallis test with subsequent Dunn’s multiple-comparisons test. All experiments were performed at least twice.

All 459 Wuhan-Hu-1 RBD-binding antibodies were subjected to a SARS-CoV-2 pseudotype neutralization assay based on the Wuhan-Hu-1 SARS-CoV-2 spike protein^5,6^. There was not a significant change in antibody potency against Wuhan-Hu-1 between 1.3 and 5 months after the second vaccine dose (half-maximal inhibitory concentration (IC_50_) of 290 versus 182 ng ml^−1^, *P* = 0.60) (Fig. 3a); however, antibody potency was greater after the third vaccine dose than 5 months after the second dose (IC_50_ of 111 versus 182 ng ml^−1^, *P* = 0.049﻿) (Fig. 3a). The overall improvement between equivalent time points after the second and third doses, from an IC_50_ of 290 ng ml^−1^ to 111 ng ml^−1^ was highly significant (*P* = 0.0023﻿) (Fig. 3a and Supplementary Table 3). Notably, the potency of antibodies isolated after the third dose, approximately 9 months (median, 266 days; range, 265–273 days) after the prime, was indistinguishable from that of antibodies isolated from convalescent vaccinated individuals 12 months after infection (*P* = 0.69﻿)^15–17^(Fig. 3a). The improved neutralizing activity was most evident among unique clones, with a marked change in IC_50_ from 323 ng ml^−1^ 1.3 months after the second vaccine dose to 67 ng ml^−1^ at 1 month after the third dose (*P* = 0.034﻿) (Fig. 3b and Supplementary Table 3). Persisting clones also showed improved neutralizing activity after the third dose (*P* = 0.043); although a trend towards improved neutralizing activity was evident among single antibodies, the difference did not reach statistical significance (Fig. 3b, Extended Data Fig. 5d and Supplementary Tables 3 and 4). In all cases, the relative potency of the antibodies isolated 1 month after the third dose was similar to that of antibodies isolated from convalescent vaccinated individuals 12 months after infection (Fig. 3a, b). Taken together, these results indicate significant improvement in the neutralizing potency against Wuhan-Hu-1 of the antibodies expressed in the memory B cell compartment 1 month after administration of a third mRNA vaccine dose compared with that observed 1.3 months after the second dose. Newly detected singlets and unique clones of expanded memory B cells detected only after the third vaccine dose accounted for most of the improvement in neutralizing activity between 5 months after the second dose and 1 month after the third dose.

## Epitopes and neutralization breadth

**Fig. 4:**
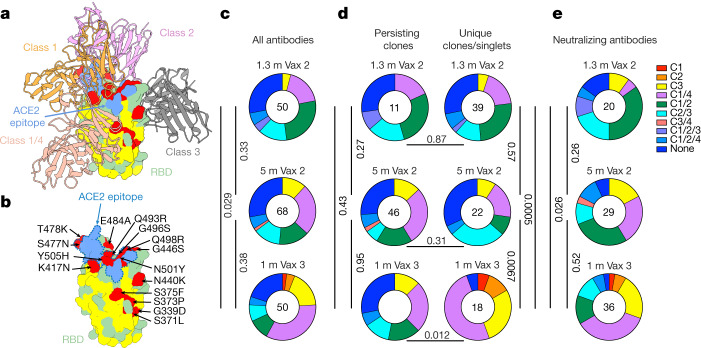
Epitope mapping. **a**, Diagram representing the binding poses of the antibodies used in BLI competition experiments on the RBD epitope. Class 1 antibody (C105, Protein Data Bank (PDB) 6XCM) is shown in orange, class 2 antibody (C144, PDB 7K90) is shown in pink, class 3 antibody (C135, PDB 7K8Z) is shown in grey and class 1/4 antibody (C118, PDB 7RKS) is shown in light coral^16,18^. The ACE2 epitope of the Omicron BA.1 variant is shown in blue. Omicron BA.1 mutations are shown in red. The most conserved residues as calculated by the ConSurf Database are shown in yellow (related to Extended Data Fig. 6). **b**, Expanded view of RBD in **a**. The ACE2 epitope of the Omicron BA.1 variant is indicated by a blue dashed line, and the Omicron BA.1 mutations are labelled. **c**–**e**, Results of epitope mapping performed by competition BLI. Pie charts show the distribution of the antibody classes among all Wuhan-Hu-1 RBD-binding antibodies (**c**), Wuhan-Hu-1 RBD-binding antibodies from persisting clones or from unique clones or singlets (**d**) or neutralizing antibodies against Wuhan-Hu-1 (**e**) obtained 1.3 and 5 months after the second vaccine dose^5,6^ and 1 month after the third vaccine dose. Statistical significance was determined using a two-tailed chi-square test.

**Extended Data Fig. 6:**
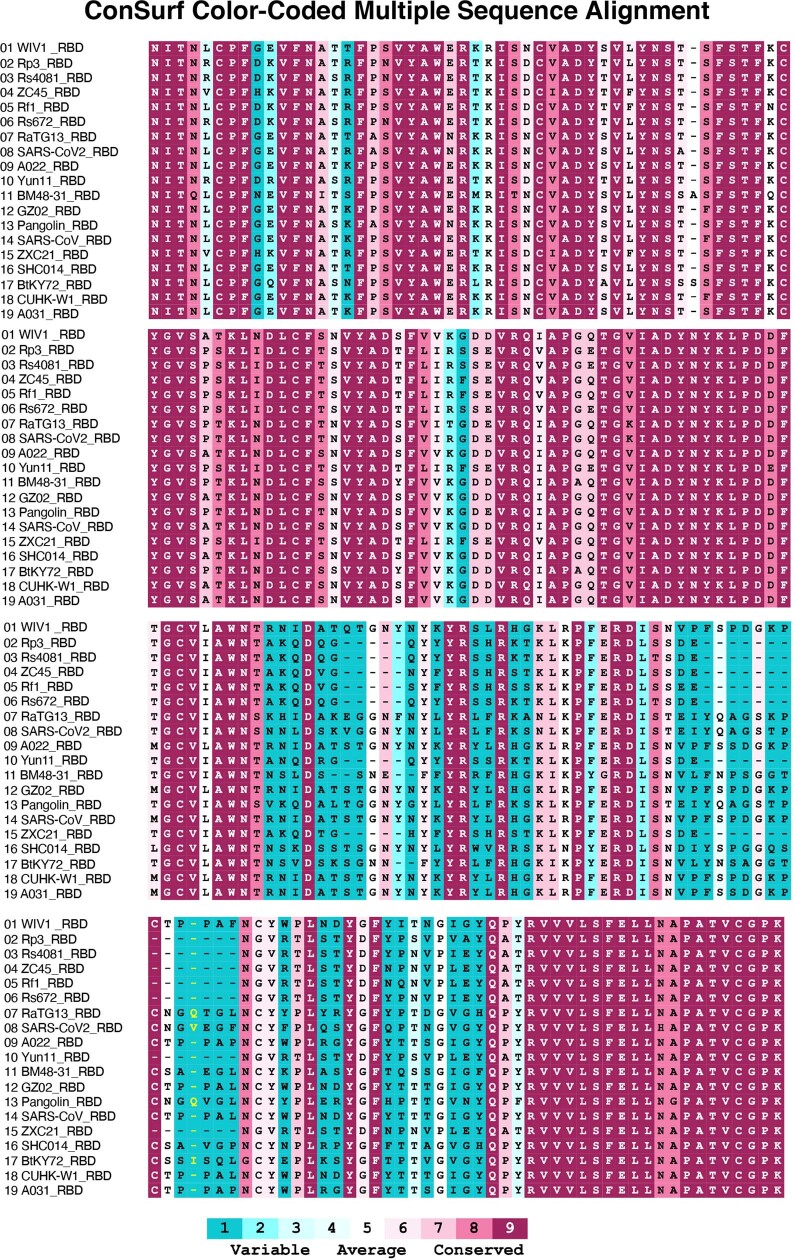
Multiple sequence alignment of RBDs. Sequences used for the alignment are the RBDs of WIV1(Bat SARS-like coronavirus WIV1, GenBank: KF367457.1), Rp3(Bat coronavirus Rp3/2004), UniprotKB:Q3I5J5), Rs4081(Bat SARS-like coronavirus isolate Rs4081, GenBank: KY417143.1), ZC45 (Bat SARS-like coronavirus ZC45, GenBank: AVP78031.1), Rf1(Bat SARS coronavirus Rf1, GenBank: DQ412042.1), Rs672(SARS coronavirus Rs_672/2006, GenBank: ACU31032.1), RaTG13(Bat coronavirus RaTG13, GenBank: QHR63300.2), SARS-CoV2 (Severe acute respiratory syndrome coronavirus 2, GenBank: MN985325.1), A022(SARS coronavirus A022, GenBank: AAV91631.1), Yun11 (Bat coronavirus Cp/Yunnan2011, GenBank: JX993988.1), BM48-31(Bat coronavirus BM48-31/BGR/2008, NCBI Reference Sequence: NC_014470.1), GZ02(SARS coronavirus GZ02, GenBank: AAS00003.1), Pangolin(Pangolin coronavirus, GenBank: QIA48632.1), SARS-CoV(Severe acute respiratory syndrome coronavirus, UniProtKB:P59594), ZXC21(Bat SARS-like coronavirus ZXC21, GenBank: AVP78042.1), SHC014(Bat SARS-like coronavirus RsSHC014, GenBank: KC881005.1), BtKY72(Severe acute respiratory syndrome-related coronavirus strain BtKY72, GenBank: KY352407.1), CUHK-W1(SARS coronavirus CUHK-W1, GenBank: AAP13567.1), and A031(SARS coronavirus A031, GenBank: AAV97988.1). Multiple sequence alignment of RBDs was processed by Clustal Omega^42^. Sequence conservation was calculated by the ConSurf Database^43^.

The majority of the anti-RBD neutralizing antibodies obtained from vaccinated individuals after the second vaccine dose belong to class 1 and class 2, which target a region overlapping the ACE2-binding site^18,19^ (Fig. 4a). These antibodies are generally more potent than class 3 and class 4 antibodies, which target the more conserved base of the RBD and do not directly interfere with ACE2 binding^17^ (Fig. 4a and Extended Data Fig. 6). Whereas class 1 and class 2 antibodies that develop early are susceptible to mutations in and around the ACE2-binding site found in many of the variants of concern, evolved versions of the same antibodies can be resistant^15,20^. According to structural information and sequence conservation among betacoronaviruses, antibodies that span class 3 or class 4 and either class 1 or class 2 are potentially broader than those spanning class 1 or class 2 alone because their epitopes include conserved sequences, and they might be more potent than antibodies from class 3 or class 4 alone because they could interfere directly with the interaction between ACE2 and the RBD (Fig. 4b and Extended Data Fig. 6).

To examine the epitopes targeted by RBD-binding antibodies after the third vaccine dose, we performed biolayer interferometry (BLI) experiments in which a preformed antibody–RBD (Wuhan-Hu-1) complex was exposed to a second antibody targeting one of the four classes of structurally defined epitopes (C105 for class 1, C144 for class 2, C135 for class 3 and C118 for class 1/4; refs. ^16,18^) (Fig. 4a). Among the 168 random RBD-binding antibodies tested, 20, 29 and 36 exhibited neutralizing activity with an IC_50_ of lower than 1,000 ng ml^−1^ from 1.3 and 5 months after the second vaccine dose and 1 month after the third dose, respectively. As might be expected, the largest group of RBD-binding antibodies obtained after the second vaccine dose belonged to class 1/2 (Fig. 4c). Although the overall distribution of classes for antibodies binding RBD did not change significantly between 1.3 and 5 months after the second dose, the relative representation of class 1 and class 2 antibodies decreased (Fig. 4c). This trend continued after the third vaccine dose, with increased representation of RBD-binding antibodies in class 1/4 and class 3, resulting in a significant difference in the epitope distribution for RBD-binding antibodies between the early time points after the second and third dose (*P* = 0.005﻿) (Fig. 4c). As expected, these differences could primarily be accounted for by the emergence of unique clones and singlets after the third vaccine dose (Fig. 4d). Similar results were found when considering neutralizing antibodies, with an initial dominance of antibodies in class 1/2 followed by increasing representation of class 1/4 and class 3 over time (Fig. 4e).

**Fig. 5:**
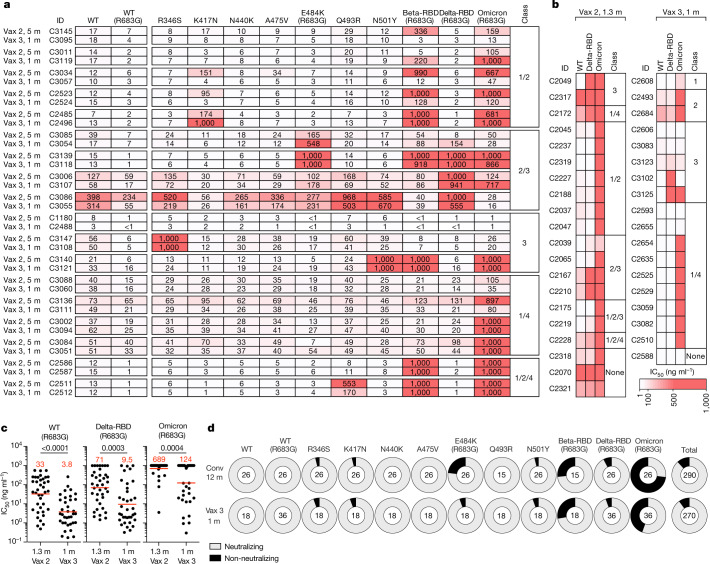
Determination of the increase in antibody breadth. **a**, **b**, Heat maps showing the IC_50_ values of clonal pairs of antibodies obtained from persisting clones at 5 months after the second vaccine dose and 1 month after the third dose (**a**) and clones and singlets found 1.3 months after the second dose^6^ and newly detected (either as a unique clone or singlet) 1 month after the third vaccine dose (**b**) against the indicated mutant and variant SARS-CoV-2 pseudoviruses. Beta-RBD and Delta-RBD indicate K417N/E484K/N501Y and L452R/T478K SARS-CoV-2 spike proteins, respectively. The heat map range from 0.1 to 1,000 ng ml^−1^ is represented by white to red. The antibody classes in **a** and **b** were determined by competition BLI. **c**, Graphs showing the neutralization activity of the antibodies shown in **a** and **b** against WT, Beta-RBD (L452R/T478K) and Omicron BA.1, comparing *n* = 38 monoclonal antibodies isolated at 1.3 months after the second vaccine dose and *n* = 36 monoclonal antibodies isolated 1 month after the third vaccine dose. Red bars and numbers indicate geometric mean values. Statistical significance was determined using the two-tailed Mann–Whitney test. *P* values are as indicated. **d**, Ring plots showing the fraction of neutralizing (IC_50_ < 1,000 ng ml^−1^) and non-neutralizing (IC_50_ > 1,000 ng ml^−1^) antibodies (represented by light grey and dark grey, respectively) for the indicated SARS-CoV-2 pseudoviruses. The numbers in the inner circles correspond to the numbers of antibodies tested.

The neutralizing breadth of the antibodies elicited by infection increases significantly after 6 months^15,17,20^, whereas no statistically significant increase in breadth has been observed 5 months after the second dose of an mRNA vaccine^6^. To determine whether neutralizing antibodies in clones that persisted from 5 months after the second dose to 1 month after the third dose develop increased breadth, we compared 18 antibody pairs that were randomly selected across participants and had measurable neutralizing activity against Wuhan-Hu-1. Neutralizing activity was measured against a panel of SARS-CoV-2 pseudoviruses harbouring RBD amino acid substitutions representative of variants, including Delta and Omicron BA.1 (Fig. 5a and Supplementary Table 5). The clonal pairs were dominated by antibodies belonging to classes 1/2, 2/3 and 3, as determined by BLI (Fig. 5a). Of the 18 antibody pairs, 15 neutralized pseudovirus carrying the Delta RBD amino acid substitutions at low antibody concentrations at both time points, with IC_50_ values of <1–154 ng ml^−1^ (Fig. 5a and Supplementary Table 5). Although the Omicron BA.1 pseudovirus showed the highest degree of neutralization resistance, 11 of the 18 antibodies isolated 1 month after the third dose neutralized this virus, 9 at an IC_50_ of below 120 ng ml^−1^ (Fig. 5a and Supplementary Table 5). Most pairs of antibodies obtained from clones persisting between 5 months after the second vaccine dose and 1 month after the third dose showed exceptionally broad neutralization, and there was little change in antibody breadth within the analysed pairs (Fig. 5a).

We extended the analysis to compare the activity of antibodies present in memory cells at 1.3 months after the second dose^6^ to that of antibodies detected uniquely 1 month after the third vaccine dose. A representative group of antibodies was selected across participants and was tested against viruses pseudotyped with spike proteins containing the RBD of Wuhan-Hu-1, Delta and Omicron BA.1 (Fig. 5b and Supplementary Table 6). We found that the proportion of Omicron BA.1-neutralizing antibodies increased from 15% after the second dose to 50% among the unique antibodies found after the third dose (*P* = 0.035, Fisher’s exact test; Fig. 5b). This increase in frequency of Omicron-neutralizing antibodies is consistent with the observation that a third vaccine dose increased the frequency of Omicron RBD-binding B cells compared with samples collected shortly before the boost^21^. Among all antibodies evaluated, the increase in breadth between the second and third vaccine doses was reflected by an increase in potency from 689 to 124 ng ml^−1^ for the IC_50_ against Omicron BA.1 (*P* = 0.0004; Fig. 5c and Supplementary Tables 5 and 6). Similar results were observed for Delta neutralization (Fig. 5c). Thus, memory B cell clones emerging after the third vaccine dose showed increasing breadth and potency against pseudoviruses representing variants that were not present in the vaccine.

Finally, we compared the neutralization breadth of antibodies elicited by the third vaccine dose, measured approximately 9 months after the prime, with that of antibodies obtained from a cohort of convalescent unvaccinated individuals 12 months after infection. The latter showed a significant increase in neutralizing breadth over time after infection^15–17^ (Fig. 5d). The two groups of antibodies were equally and remarkably broad. Moreover, 92% and 94% of the convalescent and third-dose antibodies neutralized pseudoviruses carrying the Beta RBD, and 27% and 56% neutralized Omicron BA.1, respectively. Thus, the antibodies elicited by the third dose of vaccine were at least as broad as those elicited by infection (Fig. 5d).

## Discussion

Memory B cells can develop from the germinal centre or directly from a germinal centre-independent activated B cell compartment^14^. B cells residing in germinal centres undergo multiple rounds of division, mutation and selection, whereas those in the activated compartment undergo only a limited number of divisions and carry fewer mutations^14^. Both pathways remain active throughout the immune response^22,23^. Our data indicate that the third dose of mRNA vaccines against SARS-CoV-2 expands persisting clones of memory B cells and a cohort of previously undetected clones that carry mutations indicative of germinal centre residence. The latter differ from persistent clones in that they appear to target more conserved regions of the RBD. Several different mechanisms could account for the antigenic shift, including epitope masking by high-affinity antibodies elicited by earlier vaccine doses that primarily target the less conserved RBD^18,19,24^.

Passively administered antibodies are protective against SARS-CoV-2 infection and can prevent serious disease if provided early^25–29^. The third dose of mRNA vaccines boosts plasma antibody responses to multiple SARS-CoV-2 variants, including Omicron, although the levels are insufficient to prevent breakthrough infection in many individuals^2,3^. The third dose also elicits increased numbers of memory B cells that express more potent and broader antibodies^10,21^. Although our data do not exclude the possibility that Omicron-specific memory was present before and was unaffected by the boost, others have demonstrated that the boost increases the frequency of Omicron RBD-binding memory B cells^21^. Our antibody cloning data provide a mechanistic explanation for the observed increase in Omicron-specific memory B cells after the boost. Although the memory B cells expressing more potent and broader antibodies do not appear to contribute to circulating plasma antibody levels, upon challenge with antigen in the form of a vaccine or infection, they produce large amounts of antibodies within 3–5 days^30^. Passive administration of antibodies within this same time window prevents the most serious consequences of infection^25,28,31^. Thus, rapid recall by memory T cells and a diversified and expanded memory B cell compartment are likely to be key mechanisms that contribute to the enhanced protection against severe disease by a third mRNA vaccine dose.

## Methods

### Study participants

Participants were healthy volunteers who had previously received the initial two-dose regimen of either the Moderna (mRNA-1273) or Pfizer-BioNTech (BNT162b2) mRNA vaccine against the WT (Wuhan-Hu-1) strain of SARS-CoV-2. For this study, participants were recruited for serial blood donations at The Rockefeller University Hospital in New York between 21 January and 14 December 2021. No statistical methods were used to predetermine sample size. The experiments were not randomized and the investigators were not blinded to allocation during experiments and outcome assessment. The majority of participants (*n* = 32) were follow-ups from a longitudinal cohort that we previously reported on^5,6^, while a smaller subgroup of individuals (*n* = 10) was de novo recruited for this study (for details, see Supplementary Table 1). Eligible participants (*n* = 42) were healthy adults with no history of infection with SARS-CoV-2 during or before the observation period (as determined by clinical history and confirmed through serology testing) who had received two doses of one of the two currently approved SARS-CoV-2 mRNA vaccines, Moderna (mRNA-1273) and Pfizer-BioNTech (BNT162b2). Additionally, a subgroup of individuals (*n* = 34) who had received a third vaccine dose was included. The specifics of each participant’s vaccination regimen were at the discretion of the individual and their healthcare provider, consistent with current dosing and interval guidelines, and, as such, not influenced by participation in our study. Exclusion criteria included incomplete vaccination status (defined as fewer than two doses), the presence of clinical signs and symptoms suggestive of acute infection with SARS-CoV-2, or positive reverse transcription PCR (RT–PCR) results for SARS-CoV-2 in saliva or positive COVID-19 serology. No other parameters that could lead to potential self-selection bias were used to exclude or include patients. After enrolment, participant plasma samples obtained after the third vaccine dose were tested for binding activity toward the nucleocapsid (N) protein (Sino Biological, 40588-V08B) of SARS-CoV-2. The absence of serological activity towards N protein was used to ensure a negative history for infection with SARS-CoV-2 for each participant. Participants presented to The Rockefeller University Hospital for blood sample collection and were asked to provide details of their vaccination regimen, possible side effects, comorbidities and possible COVID-19 history. Clinical data collection and management were conducted using the software iRIS by iMedRIS (version 11.02). All participants provided written informed consent before participation in the study, and the study was conducted in accordance with Good Clinical Practice. The study was performed in compliance with all relevant ethical regulations, and the protocol (DRO-1006) for studies with human participants was approved by the Institutional Review Board of The Rockefeller University. For detailed participant characteristics, see Supplementary Table 1.

### Blood sample processing and storage

Peripheral blood mononuclear cells (PBMCs) obtained from samples collected at The Rockefeller University were purified as previously reported by gradient centrifugation and were stored in liquid nitrogen in the presence of FCS and dimethylsulfoxide (DMSO)^16,17^. Heparinized plasma and serum samples were aliquoted and stored at −20 °C or lower temperatures. Before experiments, aliquots of plasma samples were heat inactivated (56 °C for 1 h) and then stored at 4 °C.

### ELISAs

ELISAs^34,35^ used to evaluate antibody binding to SARS-CoV-2 Wuhan-Hu-1 RBD were performed by coating high-binding 96-half-well plates (Corning, 3690) with 50 μl per well of a 1 μg ml^−1^ protein solution in PBS overnight at 4 °C. The plates were washed six times with washing buffer (1× PBS with 0.05% Tween-20 (Sigma-Aldrich)) and were incubated with 170 μl per well blocking buffer (1× PBS with 2% BSA and 0.05% Tween-20 (Sigma)) for 1 h at room temperature. Immediately after blocking, monoclonal antibodies or plasma samples were added to PBS and were incubated for 1 h at room temperature. Plasma samples were assayed at a 1:66 starting dilution with 10 additional threefold serial dilutions. Monoclonal antibodies were tested at a starting concentration of 10 μg ml^−1^ with 10 additional fourfold serial dilutions. Plates were washed six times with washing buffer and were then incubated with anti-human IgG, IgM or IgA secondary antibody conjugated to horseradish peroxidase (HRP) (Jackson ImmunoResearch, 109-036-088 and 109-035-129 and Sigma, A0295) in blocking buffer at a 1:5,000 dilution (IgM and IgG) or 1:3,000 dilution (IgA). Plates were developed by addition of the HRP substrate 3,3′,5,5′-tetramethylbenzidine (TMB) (ThermoFisher) for 10 min (plasma samples) or 4 min (monoclonal antibodies). The developing reaction was stopped by adding 50 μl of 1 M H_2_SO_4_, and absorbance was measured at 450 nm with an ELISA microplate reader (FluoStar Omega, BMG Labtech) with Omega and Omega MARS software for analysis. For plasma samples, a positive control (plasma from participant COV72, diluted 66.6-fold with 10 additional 3-fold serial dilutions in PBS) was added to every assay plate for normalization. The average of its signal was used for normalization of all other values on the same plate with Excel software before calculating the area under the curve using Prism version 9.3 (GraphPad). Negative controls of pre-pandemic plasma samples from healthy donors were used for validation; details of this process have previously been reported^16^. For monoclonal antibodies, the ELISA half-maximal effective concentration (EC_50_) was determined using four-parameter nonlinear regression (GraphPad Prism version 9.3). EC_50_ values above 1,000 ng ml^−1^ were considered to indicate non-binders.

### Proteins

The mammalian expression vector encoding the RBD of SARS-CoV-2 (GenBank, MN985325.1; spike protein residues 319–539) has previously been described^36^.

### SARS-CoV-2-pseudotyped reporter virus

A panel of plasmids expressing RBD-mutant SARS-CoV-2 spike proteins in the context of pSARS-CoV-2-S _Δ19_ (Wuhan-Hu-1) has previously been described^5,6,20,37^. Variant pseudoviruses resembling SARS-CoV-2 variants Beta (B.1.351), B.1.526, Delta (B.1.617.2) and Omicron BA.1 (B.1.1.529.1), as described previously^6,7,15^, were generated by introduction of substitutions using synthetic gene fragments (IDT) or overlap extension PCR-mediated mutagenesis and Gibson assembly. In particular, the following variant-specific deletions and substitutions were introduced:

Beta: D80A, D215G, L242H, R246I, K417N, E484K, N501Y, D614G, A701V

Delta: T19R, Δ156–158, L452R, T478K, D614G, P681R, D950N

Omicron BA.1: A67V, Δ69–70, T95I, G142D, Δ143–145, Δ211, L212I, ins214EPE, G339D, S371L, S373P, S375F, K417N, N440K, G446S, S477N, T478K, E484A, Q493K, G496S, Q498R, N501Y, Y505H, T547K, D614G, H655Y, H679K, P681H, N764K, D796Y, N856K, Q954H, N969H, N969K, L981F

The E484K, K417N/E484K/N501Y and L452R/T478K substitutions, as well as the deletions/substitutions corresponding to the variants of concern listed above, were incorporated into a spike protein that also included the R683G substitution, which disrupts the furin cleavage site and increases particle infectivity. The neutralizing activity against mutant pseudoviruses was compared with that against a WT SARS-CoV-2 spike sequence (NC_045512), carrying R683G where appropriate.

SARS-CoV-2-pseudotyped particles were generated as previously described^16,33^. In brief, 293T (CRL-11268) cells obtained from ATCC were transfected with pNL4-3ΔEnv-NanoLuc and pSARS-CoV-2-S_Δ19_. Particles were collected 48 h after transfection, filtered and stored at −80 °C. All cell lines used in this study were checked for mycoplasma contamination by Hoechst staining and confirmed negative.

### Pseudotyped virus neutralization assay

Fourfold serially diluted plasma samples (from healthy donors or individuals who received mRNA vaccines) or monoclonal antibodies were incubated with SARS-CoV-2-pseudotyped virus for 1 h at 37 °C. The mixture was subsequently incubated with 293T_Ace2_ cells^16^ (for all WT neutralization assays) or HT1080Ace2cl.14 cells^5^ (for all mutant panels and variant neutralization assays) for 48 h, after which cells were washed with PBS and lysed with Luciferase Cell Culture Lysis 5× reagent (Promega). NanoLuc luciferase activity in lysates was measured using the Nano-Glo Luciferase Assay System (Promega) with the Glomax Navigator (Promega). The relative luminescence units were normalized to those derived from cells infected with SARS-CoV-2-pseudotyped virus in the absence of plasma or monoclonal antibodies. The NT_50_ values for plasma or the IC_50_ and 90% inhibitory concentration (IC_90_) values for monoclonal antibodies were determined using four-parameter nonlinear regression (least-squares regression method without weighting; constraints: top = 1, bottom = 0) (GraphPad Prism).

### Biotinylation of viral protein for use in flow cytometry

Purified and Avi-tagged SARS-CoV-2 Wuhan-Hu-1 RBD was biotinylated using the BirA biotin–protein ligase kit according to the manufacturer’s instructions (Avidity) as described previously^16^. Ovalbumin (Sigma, A5503-1G) was biotinylated using the EZ-Link Sulfo-NHS-LC-Biotinylation kit according to the manufacturer’s instructions (Thermo Scientific). Biotinylated ovalbumin was conjugated to streptavidin–BV711 for single-cell sorts (BD Biosciences, 563262) or to streptavidin–BB515 for a phenotyping panel (BD Biosciences, 564453). RBD was conjugated to streptavidin–PE (BD Biosciences, 554061) and streptavidin–AF647 (BioLegend, 405237)^16^.

### Flow cytometry and single-cell sorting

Single-cell sorting by flow cytometry has previously been described^16^. In brief, PBMCs were enriched for B cells by negative selection using a pan-B cell isolation kit according to the manufacturer’s instructions (Miltenyi Biotec, 130-101-638). The enriched B cells were incubated in fluorescence-activated cell sorting (FACS) buffer (1× PBS, 2% FCS, 1 mM EDTA) with the following anti-human antibodies (all at a 1:200 dilution): anti-CD20-PECy7 (BD Biosciences, 335793), anti-CD3-APC-eFluro 780 (Invitrogen, 47-0037-41), anti-CD8-APC-eFluor 780 (Invitrogen, 47-0086-42), anti-CD16-APC-eFluor 780 (Invitrogen, 47-0168-41) and anti-CD14-APC-eFluor 780 (Invitrogen, 47-0149-42) as well as Zombie NIR (BioLegend, 423105) and fluorophore-labelled RBD and ovalbumin (Ova) for 30 min on ice. AccuCheck Counting Beads (Life Technologies, PCB100) were added to each sample according to the manufacturer’s instructions. Single CD3^−^CD8^−^CD14^−^CD16^−^CD20^+^Ova^−^RBD^−^PE^+^RBD^−^AF647^+^ B cells were sorted into individual wells of 96-well plates containing 4 μl of lysis buffer (0.5× PBS, 10 mM dithiothreitol (DTT), 3,000 units per ml of RNasin Ribonuclease Inhibitors (Promega, N2615)) per well using a FACSAria III instrument and FACSDiva software (Becton Dickinson) for acquisition and FlowJo for analysis. The sorted cells were frozen on dry ice and stored at −80 °C or were immediately used for subsequent RNA RT. For B cell phenotype analysis, in addition to the above antibodies, B cells were also stained with the following anti-human antibodies (all at a 1:200 dilution): anti-IgD-BV650 (BD, 740594), anti-CD27-BV786 (BD Biosciences, 563327), anti-CD19-BV605 (BioLegend, 302244), anti-CD71-PerCP-Cy5.5 (BioLegend, 334114), anti-IgG-PECF594 (BD, 562538), anti-IgM-AF700 (BioLegend, 314538) and anti-IgA-Viogreen (Miltenyi Biotec, 130-113-481).

### Antibody sequencing, cloning and expression

Antibodies were identified and sequenced as described previously^16,38^. In brief, RNA from single cells was reverse-transcribed (SuperScript III Reverse Transcriptase, Invitrogen, 18080-044), and the cDNA was stored at −20 °C or was used for subsequent amplification of the variable *IGH*, *IGL* and *IGK* genes by nested PCR and Sanger sequencing. Sequence analysis was performed using MacVector. Amplicons from the first PCR were used as templates for sequence- and ligation-independent cloning into antibody expression vectors. Recombinant monoclonal antibodies were produced and purified as previously described^16^.

### Biolayer interferometry

BLI assays were performed as previously described^16^. In brief, we used the Octet Red instrument (ForteBio) at 30 °C with shaking at 1,000 r.p.m. Epitope binding assays were performed with Protein A biosensor (ForteBio, 18-5010) following the manufacturer’s protocol for a classical sandwich assay as follows:

1. Sensor check: sensors immersed for 30 s in buffer alone (buffer ForteBio 18-1105)
2. Capture first antibody: sensors immersed for 10 min with antibody 1 at 10 µg ml^−1^
3. Baseline: sensors immersed for 30 s in buffer alone
4. Blocking: sensors immersed for 5 min with IgG isotype control at 10 µg ml^−1^
5. Baseline: sensors immersed for 30 s in buffer alone
6. Antigen association: sensors immersed for 5 min with RBD at 10 µg ml^−1^
7. Baseline: sensors immersed for 30 s in buffer alone
8. Association with second antibody: sensors immersed for 5 min with antibody 2 at 10 µg ml^−1^

Curve fitting was performed using the ForteBio Octet data analysis software (ForteBio).

### Computational analyses of antibody sequences

Antibody sequences were trimmed on the basis of quality and were annotated using IgBLAST version 1.14 with the IMGT domain delineation system. Annotation was performed systematically using the Change-O toolkit version 0.4.540 (ref. ^39^). The clonality of the heavy and light chains was determined using DefineClones.py implemented by Change-O version 0.4.5 (ref. ^39^). The script calculates the Hamming distance between each sequence in the dataset and its nearest neighbour. Distances are subsequently normalized to account for differences in junction sequence length, and clonality is determined on the basis of a cut-off threshold of 0.15. Heavy and light chains derived from the same cell were subsequently paired, and clonotypes were assigned according to their V and J genes using in-house R and Perl scripts. All scripts and the data used to process the antibody sequences are publicly available on GitHub (https://github.com/stratust/igpipeline/tree/igpipeline2_timepoint_v2).

The frequency distributions of human V genes in anti-SARS-CoV-2 antibodies from this study was compared with 131,284,220 heavy and light chain sequences generated by ref. ^40^ and downloaded from cAb-Rep^41^, a database of human shared B cell receptor clonotypes available at https://cab-rep.c2b2.columbia.edu/. On the basis of the 150 distinct V genes that made up the 1,650 sequences from the immunoglobulin repertoires of the five participants analysed in this study, we selected the heavy and light chain sequences from the database that were partially encoded by the same V genes and counted them according to the constant region. The frequencies shown in Extended Data Fig. 3 are relative to the source and isotype analysed. We used the two-sided binomial test to check whether the number of sequences belonging to a specific *IGHV* or *IGLV* gene in the repertoire was different according to the frequency of the same V gene in the database. The adjusted *P* values were calculated using false discovery rate correction.

Nucleotide somatic mutations and complementarity-determining region length were determined using in-house R and Perl scripts. For quantification of somatic mutations, *IGHV* and *IGLV* nucleotide sequences were aligned against their closest germline versions using IgBLAST and the number of differences was considered to be nucleotide mutations. The average number of mutations for V genes was calculated by dividing the sum of all nucleotide mutations across all participants by the number of sequences used for the analysis.

### Data presentation

The figures were arranged using Adobe Illustrator 2020 and 2022.

### Reporting summary

Further information on research design is available in the Nature Research Reporting Summary linked to this paper.

## Online content

Any methods, additional references, Nature Research reporting summaries, source data, extended data, supplementary information, acknowledgements, peer review information; details of author contributions and competing interests; and statements of data and code availability are available at 10.1038/s41586-022-04778-y.

## Supplementary information

Reporting Summary

Supplementary Table 1 Individual participant characteristics.

Supplementary Table 2 Sequences of anti-SARS-CoV-2 RBD IgG antibodies.

Supplementary Table 3 Sequences, half-maximal effective concentrations (EC_50_ values) and inhibitory concentrations (IC_50_ values) of cloned monoclonal antibodies.

Supplementary Table 4 Binding and neutralization activity of persisting clones.

Supplementary Table 5 Neutralization activity of clonal pairs isolated 5 months after the second dose and 1 month after the third dose against the mutant pseudovirus panel.

Supplementary Table 6 Neutralization activity of monoclonal antibodies isolated 1.3 months after the second dose and 1 month after the third dose against variant pseudoviruses.

## Data Availability

Data are provided in Supplementary Tables 1–6. The raw sequencing data associated with Fig. 2 have been deposited at GitHub (https://github.com/stratust/igpipeline/tree/igpipeline2_timepoint_v2). This study also used data from A Public Database of Memory and Naive B-Cell Receptor Sequences (10.5061/dryad.35ks2), PDB (6VYB and 6NB6), cAb-Rep (https://cab-rep.c2b2.columbia.edu/), the Sequence Read Archive (SRP010970) and ref. ^40^.

## Code availability

The computer code used to process the antibody sequences is available at GitHub (https://github.com/stratust/igpipeline/tree/igpipeline2_timepoint_v2).
