# Supplementary material for: Increased memory B cell potency and breadth after a SARS-CoV-2 mRNA boost
Source: Nature. 2022 Apr 21;607(7917):128–34. doi: 10.1038/s41586-022-04778-y (PMC9259484; doi:10.1038/s41586-022-04778-y)
Supplement: Supplementary file 1 — Reporting Summary [file 41586_2022_4778_MOESM1_ESM.pdf]

nature portfolio

Corresponding author(s): Theodora Hatziioannou, Paul Bieniasz, Michel C. Nussenzweig

Last updated by author(s): Apr 5, 2022

Reporting Summary

Nature Portfolio wishes to improve the reproducibility of the work that we publish. This form provides structure for consistency and transparency in reporting. For further information on Nature Portfolio policies, see our Editorial Policies and the Editorial Policy Checklist.

Statistics

For all statistical analyses, confirm that the following items are present in the figure legend, table legend, main text, or Methods section.

n/a	Confirmed	
☐	☒	The exact sample size (n) for each experimental group/condition, given as a discrete number and unit of measurement
☐	☒	A statement on whether measurements were taken from distinct samples or whether the same sample was measured repeatedly
☐	☒	The statistical test(s) used AND whether they are one- or two-sided<br>*Only common tests should be described solely by name; describe more complex techniques in the Methods section.*
☒	☐	A description of all covariates tested
☐	☒	A description of any assumptions or corrections, such as tests of normality and adjustment for multiple comparisons
☐	☒	A full description of the statistical parameters including central tendency (e.g. means) or other basic estimates (e.g. regression coefficient) AND variation (e.g. standard deviation) or associated estimates of uncertainty (e.g. confidence intervals)
☐	☒	For null hypothesis testing, the test statistic (e.g. F, t, r) with confidence intervals, effect sizes, degrees of freedom and P value noted<br>*Give P values as exact values whenever suitable.*
☒	☐	For Bayesian analysis, information on the choice of priors and Markov chain Monte Carlo settings
☒	☐	For hierarchical and complex designs, identification of the appropriate level for tests and full reporting of outcomes
☒	☐	Estimates of effect sizes (e.g. Cohen's d, Pearson's r), indicating how they were calculated

Our web collection on statistics for biologists contains articles on many of the points above.

Software and code

Policy information about availability of computer code

Data collection	IRIS by iMedRIS version 11.02 for clinical data collection and management; BD FACSDiva Software Version 8.0.2 for flow sorting; Glomax Navigator Promega V.3 for neutralization assays; Omega 5.11 by BMG Labtech was used for Elisa Assays.
Data analysis	FlowJo 10.6.2 for FACS analysis; GraphPad Prism 9.3; Microsoft Excel 16.5.7; MacVector 17.5.4 for sequence analysis; Omega MARS V2.10 by BMG Labtech for luminometer; Glomax Navigator V.3 from Promega, Fortebio Octet Data analysis software Version 10.0, Adobe Illustrator 2020 and 2022, BBDuk for sequencing read processing, scripts and the data used to process antibody sequences are available on GitHub (https://github.com/stratust/igpipeline/tree/igpipeline2_timepoint_v2).

For manuscripts utilizing custom algorithms or software that are central to the research but not yet described in published literature, software must be made available to editors and reviewers. We strongly encourage code deposition in a community repository (e.g. GitHub). See the Nature Portfolio guidelines for submitting code & software for further information.

Data

Policy information about availability of data

All manuscripts must include a data availability statement. This statement should provide the following information, where applicable:

- Accession codes, unique identifiers, or web links for publicly available datasets
- A description of any restrictions on data availability
- For clinical datasets or third party data, please ensure that the statement adheres to our policy

Data are provided in Supplementary Tables 1-6. The raw sequencing data associated with Figure 2 have been deposited at Github (https://github.com/stratust/igpipeline/tree/igpipeline2_timepoint_v2). This study also uses data from “A Public Database of Memory and Naive B-Cell Receptor Sequences” (https://

doi.org/10.5061/dryad.35ks2), PDB (6VYB and 6NB6), cAb-Rep (https://cab-rep.c2b2.columbia.edu/), Sequence Read Archive (accession SRP010970), and from "High frequency of shared clonotypes in human B cell receptor repertoires" (https://doi.org/10.1038/s41586-019-0934-8).

Field-specific reporting

Please select the one below that is the best fit for your research. If you are not sure, read the appropriate sections before making your selection.

☒ Life sciences ☐ Behavioural & social sciences ☐ Ecological, evolutionary & environmental sciences

For a reference copy of the document with all sections, see nature.com/documents/nr-reporting-summary-flat.pdf

Life sciences study design

All studies must disclose on these points even when the disclosure is negative.

Sample size	Sample size of 42 individuals was based on how many study participants could be recruited for serial blood donations at the Rockefeller University Hospital in New York between January 21, 2021, and December 14, 2021. The majority of participants (n=32) were follow-ups from a longitudinal cohort that we previously reported on (Wang et al 2021 and Cho et al 2021), while a smaller subgroup of individuals (n=10) was de novo recruited for this study (for details see Supplementary Table 1).
Data exclusions	No data were excluded from the analysis.
Replication	All experiments successfully performed at least twice.
Randomization	This is not relevant as this is an observational study.
Blinding	This is not relevant as this is an observational study.

Reporting for specific materials, systems and methods

We require information from authors about some types of materials, experimental systems and methods used in many studies. Here, indicate whether each material, system or method listed is relevant to your study. If you are not sure if a list item applies to your research, read the appropriate section before selecting a response.

Materials & experimental systems

n/a	Involved in the study	
☐	☒	Antibodies
☐	☒	Eukaryotic cell lines
☒	☐	Palaeontology and archaeology
☒	☐	Animals and other organisms
☐	☒	Human research participants
☒	☐	Clinical data
☒	☐	Dual use research of concern

Methods

n/a	Involved in the study	
☒	☐	ChIP-seq
☐	☒	Flow cytometry
☒	☐	MRI-based neuroimaging

Antibodies

Antibodies used

1. Mouse anti-human CD20-PECy7 (BD Biosciences, 335793), clone L27
2. Mouse anti-human CD3-APC-eFluro 780 (Invitrogen, 47-0037-41), clone OKT3
3. Mouse anti-human CD8-APC-421eFluro 780 (Invitrogen, 47-0086-42), clone OKT8
4. Mouse anti-human CD16-APC-eFluro 780 (Invitrogen, 47-0168-41), clone eBioCB16
5. Mouse anti-human CD14-APC-eFluro 780 (Invitrogen, 47-0149-4), clone 61D3
6. Zombie NIR (BioLegend, 423105)
7. Mouse anti-IgD-BV650 (BD Biosciences, 740594), clone IA6-2
8. Mouse anti-CD27-BV786 (BD Biosciences, 563327), clone L128
9. Mouse anti-human CD19-BV605 (Biolegend, 302244), clone HIB19
10. Mouse anti-human CD71-PerCPCy5.5 (Biolegend, 334114), clone CY1G4
11. Mouse anti-human IgG-PECF594 (BD Bioscience, 562538), clone G18-145
12. Mouse anti-human IgM-AF700 (Biolegend, 314538), clone MHM-88
13. Mouse anti-human IgA-VioGreen (Miltenyi Biotec, 130-113-481), clone IS11-8E10
14. Peroxidase Goat anti-Human IgG Jackson Immuno Research 109-036-088
15. Peroxidase Goat anti-Human IgM Jackson Immuno Research 109-035-129
16. Peroxidase Goat anti-Human IgA Sigma A0295
17. Streptavidin-BV711 (BD biosciences Cat#563262)
18. Streptavidin-PE (BD biosciences Cat#554061)

19. Streptavidin-AF647 (Biolegend Cat#405237)

Validation

All antibodies are commercially available and validated by manufacturers. Additionally, information can be found on product website, listed below.
1. https://www.bdbiosciences.com/en-us/products/reagents/flow-cytometry-reagents/clinical-discovery-research/single-color-antibodies-ruo-gmp/pe-cy-7-mouse-anti-human-cd20.335793
2. https://https://www.biolegend.com/en-us/products/zombie-nir-fixable-viability-kit-8657www.thermofisher.com/antibody/product/CD3-Antibody-clone-OKT3-Monoclonal/47-0037-42
3. https://www.thermofisher.com/antibody/product/CD8a-Antibody-clone-OKT8-OKT-8-Monoclonal/47-0086-42
4. https://www.thermofisher.com/antibody/product/CD16-Antibody-clone-eBioCB16-CB16-Monoclonal/47-0168-42
5. https://www.thermofisher.com/antibody/product/CD14-Antibody-clone-61D3-Monoclonal/47-0149-42
6. https://www.biolegend.com/en-us/products/zombie-nir-fixable-viability-kit-8657
7. https://www.bdbiosciences.com/en-ca/products/reagents/flow-cytometry-reagents/research-reagents/single-color-antibodies-ruo/bv650-mouse-anti-human-igd.740594
8. https://www.bdbiosciences.com/en-ca/products/reagents/flow-cytometry-reagents/research-reagents/single-color-antibodies-ruo/bv786-mouse-anti-human-cd27.563327
9. https://www.biolegend.com/en-us/products/brilliant-violet-605-anti-human-cd19-antibody-8483?GroupID=BLG5913
10. https://www.biolegend.com/en-us/products/percp-cyanine5-5-anti-human-cd71-antibody-9387?GroupID=BLG4836
11. https://www.bdbiosciences.com/en-us/products/reagents/flow-cytometry-reagents/research-reagents/single-color-antibodies-ruo/pe-cf594-mouse-anti-human-igg.562538
12. https://www.biolegend.com/fr-lu/products/alexa-fluor-700-anti-human-igm-antibody-12507
13. https://www.miltenyibiotec.com/US-en/products/iga-antibody-anti-human-is11-8e10.html#gref
14. https://www.jacksonimmuno.com/catalog/products/109-036-088
15. https://www.jacksonimmuno.com/catalog/products/109-035-129
16. https://www.sigmaaldrich.com/US/en/product/sigma/a0295
17.https://www.bdbiosciences.com/en-us/products/reagents/flow-cytometry-reagents/research-reagents/single-color-antibodies-ruo/bv711-streptavidin.563262
18.https://www.bdbiosciences.com/en-us/products/reagents/flow-cytometry-reagents/research-reagents/single-color-antibodies-ruo/pe-streptavidin.554061
19.https://www.biolegend.com/en-gb/products/alexa-fluor-647-streptavidin-9305?GroupID=GROUP23

Eukaryotic cell lines

Policy information about cell lines

Cell line source(s)

293T (ATCC CRL-11268)
293T/ACE2* (Robbiani, D. et al. Nature 584, doi.org/10.1038/s41586-020-2456-9)
HT1080/ACE2.cl14 (Schmidt, F. et al. J Exp Med 217, doi:10.1084/jem.20201181)

Authentication

Not authenticated after purchase from ATCC.

Mycoplasma contamination

All cell lines were tested negative for mycoplasma contamination as assessed by Hoechst staining.

Commonly misidentified lines
(See ICLAC register)

No commonly misidentified cell lines were used.

Human research participants

Policy information about studies involving human research participants

Population characteristics

Participants were healthy volunteers who had previously received the initial two-dose regimen of either the Moderna (mRNA-1273) or Pfizer-BioNTech (BNT162b2) mRNA vaccines against the wildtype (Wuhan-Hu-1) strain of the severe acute respiratory syndrome coronavirus 2 (SARS-CoV-2). Particpants were 34 (23-78) years old (median (range)), 22 out of 42 participants were female. 8 participants received the Moderna (mRNA-1273) and 34 received the Pfizer-BioNTech (BNT162b2) vaccine.

Recruitment

participants were recruited for serial blood donations at the Rockefeller University Hospital in New York between January 21, 2021, and December 14, 2021. The majority of participants (n=32) were follow-ups from a longitudinal cohort that we previously reported on (Wang et al. 2021 and Cho et al., 2021) while a smaller subgroup of individuals (n=10) was de novo recruited for this study (for details see Supplementary Table 1). Eligible participants (n=42) were healthy adults with no history of infection with SARS-CoV-2 during or prior to the observation period (as determined by clinical history and confirmed through serology testing) who had received two doses of one of the two currently approved SARS-CoV-2 mRNA vaccines, Moderna (mRNA-1273) or Pfizer-BioNTech (BNT162b2), and this also included a subgroup of individuals (n=33) who had received a third vaccine dose. The specifics of each participant's vaccination regimen were at the discretion of the individual and their health care provider consistent with current dosing and interval guidelines and, as such, not influenced by their participation in our study. Exclusion criteria included incomplete vaccination status (defined as less than 2 doses), presence of clinical signs and symptoms suggestive of acute infection with or a positive reverse transcription polymerase chain reaction (RT-PCR) results for SARS-CoV-2 in saliva, or a positive (coronavirus disease 2019) COVID-19 serology. No other parameters that could lead to potential self-selection bias were used to exclude or include patients. Participants presented to the Rockefeller University Hospital for blood sample collection and were asked to provide details of their vaccination regimen, possible side effects, comorbidities and possible COVID-19 history.

Ethics oversight	The study was performed in compliance with all relevant ethical regulations and the protocol (DRO-1006) for studies with human participants was approved by the Institutional Review Board of the Rockefeller University.

Note that full information on the approval of the study protocol must also be provided in the manuscript.

Flow Cytometry

Plots

Confirm that:

☒ The axis labels state the marker and fluorochrome used (e.g. CD4-FITC).

☒ The axis scales are clearly visible. Include numbers along axes only for bottom left plot of group (a 'group' is an analysis of identical markers).

☒ All plots are contour plots with outliers or pseudocolor plots.

☒ A numerical value for number of cells or percentage (with statistics) is provided.

Methodology

Sample preparation	Whole blood samples were obtained from study participants recruited through Rockefeller University Hospital. Peripheral blood mononuclear cells (PBMCs) were separated by Ficoll gradient centrifugation. Prior to sorting, PBMCs were enriched for B cells using a Miltenyi Biotech pan B cell isolation kit (cat. no. 130-101-638) and LS columns (cat. no. 130-042-401).
Instrument	FACS Aria III (Becton Dickinson)
Software	BD FACSDiva Software Version 8.0.2 and FlowJo 10.6.2
Cell population abundance	Sorting efficiency ranged from 40% to 80%. This is calculated based on the number of IgG-specific antibody sequences that could be PCR-amplified successfully from single sorted cells from each donor.
Gating strategy	Cells were first gated for lymphocytes in FSC-A (x-axis) versus SSC-A (y-axis). We identify single cells in FSC-A versus FSC-H, and then SSC-A versus SSC-W. We then select for CD20+ Dump- B Cells in dump (anti-CD3-eFluro 780, anti-CD16-eFluro 780, anti-CD8-eFluro 780, anti-CD14-eFluro 780, Zombie NIR) versus CD20 (anti-CD20-PE-Cy7); dump-negative was considered to be signal less than 250, and CD20-positive was taken to be signal greater than 100. We then gate for Ova- B cells in FSC-A versus Ova-BV711; Ova-negative was considered to be all cells with signal less than 100. Select for Sars-CoV-2 RBD double-positive cells in RBD PE versus RBD AlexaFluor 647; this gate was made along the 45° diagonal, above 1000 on both axes.

☒ Tick this box to confirm that a figure exemplifying the gating strategy is provided in the Supplementary Information.
